# Effects of a 7-Day Meditation Retreat on the Brain Function of Meditators and Non-Meditators During an Attention Task

**DOI:** 10.3389/fnhum.2018.00222

**Published:** 2018-06-11

**Authors:** Elisa H. Kozasa, Joana B. Balardin, João Ricardo Sato, Khallil Taverna Chaim, Shirley S. Lacerda, João Radvany, Luiz Eugênio A. M. Mello, Edson Amaro Jr.

**Affiliations:** ^1^Hospital Israelita Albert Einstein, São Paulo, Brazil; ^2^Center of Mathematics, Computation and Cognition, Universidade Federal do ABC, Santo André, Brazil; ^3^Department of Physiology, Universidade Federal de São Paulo, São Paulo, Brazil

**Keywords:** meditation, retreat, fMRI, attention, Stroop task

## Abstract

Meditation as a cognitive enhancement technique is of growing interest in the field of health and research on brain function. The Stroop Word-Color Task (SWCT) has been adapted for neuroimaging studies as an interesting paradigm for the understanding of cognitive control mechanisms. Performance in the SWCT requires both attention and impulse control, which is trained in meditation practices. We presented SWCT inside the MRI equipment to measure the performance of meditators compared with non-meditators before and after a meditation retreat. The aim of this study was to evaluate the effects of a 7-day Zen intensive meditation training (a retreat) on meditators and non-meditators in this task on performance level and neural mechanisms. Nineteen meditators and 14 non-meditators were scanned before and after a 7-day Zen meditation retreat. No significant differences were found between meditators and non-meditators in the number of the correct responses and response time (RT) during SWCT before and after the retreat. Probably, due to meditators training in attention, their brain activity in the contrast incongruent > neutral during the SWCT in the anterior cingulate, ventromedial prefrontal cortex/anterior cingulate, caudate/putamen/pallidum/temporal lobe (center), insula/putamen/temporal lobe (right) and posterior cingulate before the retreat, were reduced compared with non-meditators. After the meditation retreat, non-meditators had reduced activation in these regions, becoming similar to meditators before the retreat. This result could be interpreted as an increase in the brain efficiency of non-meditators (less brain activation in attention-related regions and same behavioral response) promoted by their intensive training in meditation in only 7 days. On the other hand, meditators showed an increase in brain activation in these regions after the same training. Intensive meditation training (retreat) presented distinct effects on the attention-related regions in meditators and non-meditators probably due to differences in expertise, attention processing as well as neuroplasticity.

## Introduction

There is growing interest in meditation as a way to improve cognitive performance and emotional balance. Even though researches suggest that meditation modulates brain activities associated with cognitive control, the neural mechanisms underlying the benefits of meditation are not completely understood. It might be, for some authors, a “top–down” regulation strategy, while for others a “bottom–up”. Other studies suggest that meditation practice is associated with changes in structures working synergistically to enhance self-regulation: the anterior cingulate cortex, insula, temporo-parietal junction, fronto-limbic network and default mode network (DMN; Hölzel et al., 2011; Vago and Silbersweig, 2012; Chiesa et al., 2013; Sun et al., 2015; Tang et al., 2015).

A meta-analysis reviewed 78 functional neuroimaging studies and revealed that despite of dissociable patterns for different meditation categories (such as focused attention (FA), open monitoring, mantra and compassion/loving-kindness meditation practices) brain regions, involved in cognitive control (e.g., impulse control), proprioception and motor regulation are commonly presented in all these styles: insula, dorsal anterior cingulate, frontopolar, pre/supplementary motor cortices (Fox et al., 2016).

Meditation practice may also change brain structure. In another meta-analysis, the authors found brain differences in meditators compared with non-meditators or naïve meditators, in frontopolar and orbitofrontal cortex, insula, hippocampus, anterior and mid cingulate cortex, superior longitudinal fasciculus and corpus callosum. These regions are implicated in meta, interoceptive and exteroceptive awareness, memory, emotional regulation, intra and interhemispheric communication between brain regions (Fox et al., 2014).

There is evidence that even a short-term meditation practice may alter large-scale brain networks. In a study applying multivariate pattern analysis to resting-state fMRI (rsfMRI) data, whole brain rsfMRI was performed. Participants received 2 weeks of mindfulness meditation 30 min per session. Classifiers were able to differentiate (72% accuracy) patterns of connectivity from before vs. after the training. After training, an increase in functional connections were detected, involving the neural circuitry related to attention, cognitive and affective processing among others (Tang et al., 2017).

Cognitive control, one of the main abilities developed through meditation practice, has been studied extensively using tasks such as the Stroop Word-Color Task (SWCT), flanker and spatial conflict task. The SWCT has been adapted for fMRI studies as an interesting paradigm for the understanding of cognitive control mechanisms. The simultaneous presentation of a painted written word stimulus will be congruent when the color and word stimuli are the same, e. g., “red” written in the color red or incongruent, e.g., “green” written in red, or neutral, e.g., the word “pencil” in red. During the task, the participant has to choose the color inhibiting the impulse of reading the word (Peterson et al., 1999). The SWCT requires both attention and impulse control, characteristics developed through meditation, therefore an interesting task to evaluate the effects of this practice.

A conflict resolution task, such as the SWCT, requires working memory, attention to stimuli, target-stimulus comparison, response preparation, and response initiation. Neuroimaging studies typically identify brain regions engaged in conflict processing, which includes the dorsolateral prefrontal cortex (DLPFC), anterior cingulate cortex/pre-supplementary motor area (ACC/pSMA), dorsal premotor cortex (dPMC), anterior insular cortex (AIC), inferior frontal junction (IFJ) and posterior parietal cortex (PPC; Ridderinkhof et al., 2004; Cole and Schneider, 2007; Roberts and Hall, 2008). The ACC plays a central role in conflict monitoring (Botvinick et al., 2001), while the AIC, inferior frontal gyrus (IFG) and pSMA do so during inhibitory control tasks (Meffert et al., 2016). Considering the overlap of brain regions involved in meditation and SWCT this task is eligible to study possible brain changes due to this practice.

The SWCT has been tested to evaluate the performance of meditators compared with non-meditators, or different groups before and after meditation training. Meditation experience is associated with reduced interference during the Stroop task (Chan and Woollacott, 2007). Better attentional performance was found in meditators compared with a control group of non-meditators in the Stroop Task (Moore and Malinowski, 2009). In a more recent study these authors evaluated elders who participated in 8-week mindful breath awareness training or in brain training exercises, and they completed a modality of Stroop task which measures attentional and emotional control before and after the interventions. The mindful breath group presented significant behavioral and electrophysiological responses related to this task performance (Malinowski et al., 2017).

With the same behavioral performance level, non-meditators showed an increased pattern of brain activation relative to meditators in the right medial frontal gyrus, middle temporal gyrus, lentiform nucleus, precentral gyrus and postcentral gyrus during the SWCT incongruent compared to congruent conditions, suggesting that meditation practice may be associated with increased brain efficiency (Kozasa et al., 2012).

Taking the previous literature into account, the aim of this study was to evaluate the effects of a 7-day Zen meditation retreat on meditators and non-meditators in an attention and impulse control task, the SWCT. We hypothesized that the effects of a meditation retreat would change the brain activation in meditators and non-meditators in the anterior cingulate, the insula, the striatum, the temporal and the precentral gyrus. We also hypothesized that non-meditators would reduce their brain activation from before to after the meditation retreat, while meditators would keep the same level of brain activation from before to after the retreat.

## Materials and Methods

### Study Design

This is a case-control study to evaluate the effects of an intervention, a meditation retreat, on meditators and non-meditators.

### Participants

Nineteen meditators (five men; mean age 43.26 ± 10.97)—who have been practicing meditation for at least 3 years, three times a week, 30 min minimum each session from different meditation traditions (zen, Kriya yoga and mindfulness of breathing)—and 14 non-meditators (three men; mean age 46.79 ± 8.27), with high level of education (18 meditators and 12 non-meditators with at least university degree), were scanned during fMRI in a SWCT block design paradigm, before and after a 7-day Zen meditation retreat (*Sesshin*). They were recruited from meditation centers and mailings sent to groups interested in the subject of meditation. A physician and a neuropsychologist were involved in the selection process, and after clinical evaluation and tests, the participants who presented neurological or psychiatric disorders or were not able to undergo MRI exams, were excluded.

### Ethics Approval

The Hospital Israelita Albert Einstein Ethics Committee approved the study (approval number: 0214.0.028.000-07). The procedures were in accordance with the Declaration of Helsinki (1989), and the participants signed the informed consent before starting the tests.

### Zen Meditation

Zen meditation trains attention by focusing body and mind in a mindful way. The purpose is to be present in the here and now. It emphasizes the direct experience of the ordinary activities (Austin, 2013).

The attentional family of meditation practices include FA and open monitoring practices (OM). Narrow attentional scope and one-pointed concentration on a single object (e.g., paying attention to the breath) characterizes FA, while OM involves the development of meta-awareness, without getting involved in one specific object. Instead, the attention involves whatever perceptions, emotions and thoughts in the field of awareness. Zen meditation is an OM technique and its challenge is to overcome mind-wandering or distractions, which happen when the attention is directed away from the field of awareness and the monitoring process (Dahl et al., 2015).

### 7-Day Zen Meditation Retreat (*Sesshin*)

During meditation sessions (*zazen*), participants were instructed to sit in an upright position, to avoid movements and just observe sensations, thoughts and any other experience (Austin, 2013). The eyes were open during the meditation practice.

Sitting meditation sessions (Zazen-Shikantaza) were alternated with slow walking meditation (*kinhin*). Participants were instructed to keep awareness and silence during all retreat time, even during meals and any other activity. The duration of the activities was almost 12 h a day. The retreat was conducted by the head of a Zen Center with decades of experience and who had received training in Japan for more than 15 years (Table 1).

**Table 1:** Daily retreat program activities.

Morning	Afternoon	Evening
05:10 – Wake up	12:00 – Lunch	18:20 – Reading texts
05:30 – Zazen	01:00 – Break	18:30 – Dinner
06:00 – Kinhin	15:00 – Coffee-break	19:30 – Zazen
06:10 – Zazen	15:30 – Zazen	20:00 – Kinhin
06:40 – Kinhin	16:00 – Kinhin	20:10 – Zazen
06:50 – Reading texts	16:10 – Zazen	20:40 – Kinhin
07:20 – Breakfast	16:40 – Kinhin	20:40 – Zazen
08:15 – Break	16:50 – Zazen	21:00 – Tea time
08:30 – Cleaning the place	17:20 – Yoga	21:30 – End of activities
09:00 – Yoga		
10:30 – Zazen		
11:00 – Kinhin		
11:10 – Zazen		
11:40 – Kinhin		
11:50 – Reading texts		

### Image Acquisition

The SWCT task was adapted to fMRI and the participants were evaluated in a block design. Image acquisition was performed in a 3.0T MR system—Siemens Tim Trio, 12ch head coil, with visual stimuli presentation via goggles (NNL systems) and participant responses were synchronized (NNL systems^1^). The fMRI acquisition was based on T2*-weighted echo planar (EPI) images for the whole brain. Acquisition parameters were EPI GRE T2*-BOLD PACE: TR = 2000 ms, TE = 50 ms, 32 slices, 3.3 mm of slice thickness, 0.5 mm of interslice gap, FOV = 200 mm, and matrix 64 × 64, 3 mm^3^ voxels, with 180 volumes. First four EPI volumes were discarded to prevent the period of stabilization of the signal. Each word stimulus was presented for 1 s interspersed by a fixation cross for 1 s. Total task time: 6 min.

### fMRI Procedure

**Figure 1:**
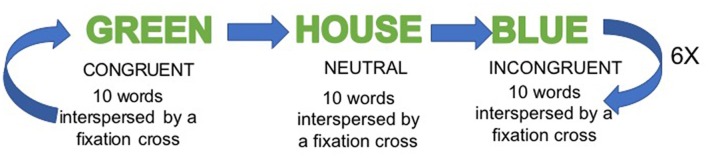
Stroop Word-Color Task (SWCT) fMRI paradigm.

The SWCT task was administered over 6 min using a block design incorporating alternating incongruent, congruent and neutral conditions. The neutral condition was considered as the baseline condition. There were six blocks of each condition, with 10-word stimuli per block. Within each one of the blocks, each stimulus (i.e., word) was presented on a black background (duration 1000 ms), followed by a fixation cross (duration 1000 ms). Participants were instructed to communicate the color (red, blue or green) of single words presented in three conditions: congruent CON (same word-color), neutral NEU (words unrelated to any color) and incongruent INC (different word-color) by pressing one of three buttons. Blocks were presented in the sequence CON, NEU and INC (Figure 1).

### Image Processing

The fMRI data processing was carried out using FSL^2^ (Smith et al., 2004). The volumes were processed by movement correction (MCFLIRT), spatial smoothing (FWHM = 5 mm) and spatial normalization to standard space (affine, 12 DoF). The individual activation maps were produced using the general linear model (GLM) with FILM routines, which is based on semi-parametric estimation of residuals autocorrelation (Woolrich et al., 2001). The model consisted of two regressors of interest (i.e., incongruent and congruent), which modeled the 20 s block duration, convolved with a gamma hemodynamic response function. At the second-level analysis, group comparisons were obtained using the mixed-effects model. We determined the contrasts of hemodynamic response function beta estimates for the incongruent relative to the congruent, as well as for the incongruent relative to the neutral baseline condition. At the group level, using the mixed-effects model, the group*time interaction was examined using a 2-way repeated measures ANOVA applying the within-subject factor TIME (pre- vs. post-training) and the between-subject factor GROUP (meditators vs. non-meditators). All the statistical images were thresholded by using Gaussian random field-based cluster inference with a threshold of *Z* > 1.96 at the voxel level and a corrected cluster significance threshold of *Z* < 0.05. Group × time interaction was also examined for behavioral SWCT measures (Stroop effect and number of correct responses).

## Results

### Stroop Task Behavioral Results

No significant effects of response time (RT) neither group × time interaction was observed in the RT to the incongruent condition in the SWCT. We detected no significant differences between group × time interaction and across time (*p* = 0.008) in the average of correct responses to the incongruent condition (Table 2).

**Table 2:** Stroop task time reaction and correct responses.

	Meditators (*n* = 18)		Non-Meditators (*n* = 13)				
	Pre	Post	Pre	Post	Time effect	Group effect	Time × Group effect
	Average (SD)	Average (SD)	Average (SD)	Average (SD)	*p*	*p*	*p*
Time reaction							
Congruent	710.37 (111.99)	716.01 (103.85)	707.25 (105.94)	718.78 (85.19)	0.288	0.996	0.713
Neutral	733.32 (117.50)	770.97 (126.77)	729.63 (101.78)	806.53 (88.66)	0.001	0.674	0.084
Incongruent	809.59 (135.48)	826.50 (120.34)	810.20 (122.67)	829.05 (112.64)	0.059	0.971	0.916
Correct responses							
Congruent	9.86 (0.14)	9.83 (0.18)	9.72 (0.43)	9.76 (0.27)	0.969	0.171	0.597
Neutral	9.83 (0.24)	9.86 (0.18)	9.72 (0.28)	9.74 (0.29)	0.592	0.142	0.886
Incongruent	9.43 (0.58)	9.48 (0.42)	9.63 (0.34)	9.57 (0.53)	0.134	0.964	0.575

*Legend: repeated measures MANOVA test*.

### Stroop Task Imaging Results

**Figure 2:**
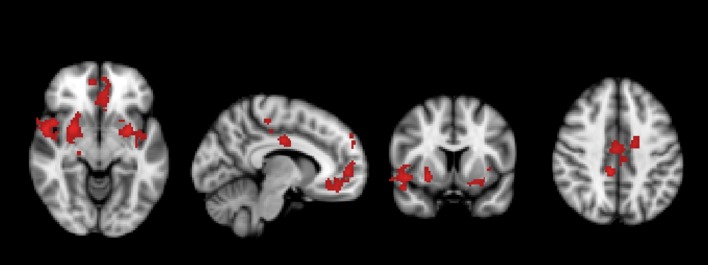
Regions in which a significant group × time interaction was observed in the comparison between meditators and non-meditators in the contrast incongruent (INC) > neutral (NEU) before and after a meditation retreat.

**Figure 3:**
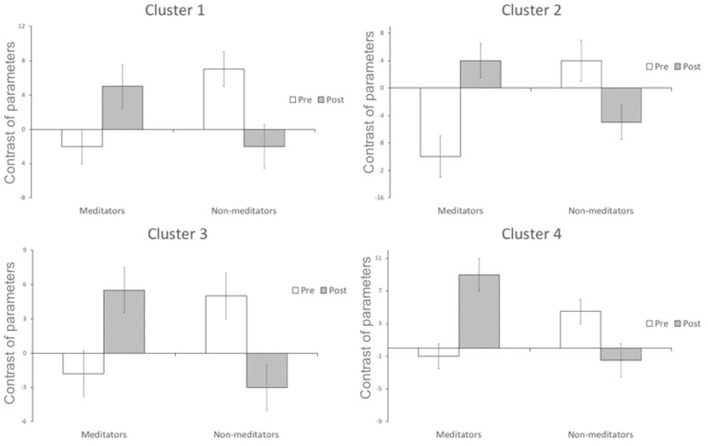
Change in functional activation in the regions of significant group × session interaction (mean and standard-error bars) showing that after the retreat, there is a decrease in activation in the non-meditator group, and an increase in the meditator group. Cluster 1 encompasses caudate nucleus, temporal lobe, putamen and insula; cluster 2 encompasses putamen, temporal lobe and caudate nucleus; cluster 3 encompasses anterior cingulate, frontal lobe, paracingulate; cluster 4 encompasses posterior cingulate, paracingulate, anterior cingulate, callosal body, frontal lobe and juxtapositional lobule cortex.

A significant group*time interaction on brain activation in the contrast INC > NEU was observed in clusters encompassing the anterior cingulate/ventromedial prefrontal cortex, putamen/pallidum/caudate/temporal lobe (center), insula/putamen/temporal lobe (right) and the posterior cingulate (Table 3, Figure 2). Plots showing the mean magnitude estimates of activity in the significant clusters for each session indicate the nature of the interaction effect (Figure 3). In non-meditators, all regions presented higher activation before the retreat than thereafter. In meditators, the BOLD response showed the opposite pattern.

**Table 3:** Regions with a significant group × time interaction observed in the comparison between meditators and non-meditators in the contrast INC > NEU before and after a meditation retreat.

Contrast	Cluster	Region	Side	*Z*	MNI coordinates			Cluster size	Cluster *p*-values
					*x*	*y*	*z*		
Incong > neutral	Cluster 1	Caudate nucleus	R	3.72	24	−16	22	1508	0.00144
		Temporal Lobe	R	3.39	52	4	−6		
		Temporal Lobe	R	3.32	50	10	−18		
		Putamen	R	3.29	26	4	−8		
		Insula	R	3.05	48	8	−8		
		Temporal Lobe	R	3.04	52	−6	−18		
	Cluster 2	Putamen	L	3.96	−24	−8	18	1327	0.00362
		Putamen	L	3.77	−26	−8	10		
		Temporal Lobe	L	3.45	−28	2	−16		
		Putamen	L	3.44	−32	−2	2		
		Putamen	L	3.21	−28	2	−8		
		Caudate nucleus	L	3.15	−14	28	4		
	Cluster 3	Anterior Cingulate	L	3.21	0	30	4	1246	0.00554
		Frontal Lobe	L	3.17	0	30	−2		
		Frontal Lobe	L	3.15	2	20	−20		
		Paracingulate	L	3.08	−6	52	2		
		Paracingulate	L	3.07	−6	40	−10		
		Paracingulate	L	3.04	−4	56	12		
	Cluster 4	Posterior Cingulate		3.22	6	−34	36	868	0.0455
		Paracingulate	R	3.21	8	−34	40		
		Anterior Cingulate	L	3.06	−10	−12	34		
		Callosal Body	R	3	−18	−6	40		
		Frontal Lobe	L	2.96	−16	−8	48		
		Juxtapositional Lobule Cortex	L	2.95	−14	−4	48		

## Discussion

The objective of this study was to evaluate the effects of a 7-day Zen meditation retreat on meditators and non-meditators in an attention and inhibition task—the SWCT.

Zen belongs to the attentional family group of meditation practices, characterized by “the systematic training of the capacity to intentionally initiate, direct, and/or sustain attentional processes while strengthening the capacity to be aware of the processes of thinking, feeling, and perceiving” (Dahl et al., 2015). In Zen, it is emphasized the practice of attention during daily activities. The practitioner is trained to keep awareness on the impulses and be able to regulate the responses to external and internal stimuli (Austin, 2013).

### Within Group Analysis

In the within group analysis, comparing only non-meditators before and after the retreat, clusters encompassing posterior cingulate gyrus, caudate, occipital lobe and frontal pole presented reduced activation after the retreat; comparing only meditators before and after the retreat, clusters encompassing middle frontal gyrus, parietal lobe, IFG and superior parietal lobule showed reduced activation after the retreat compared to before (Supplementary Figure S1). These regions are commonly activated during the SWCT (Ridderinkhof et al., 2004; Cole and Schneider, 2007; Roberts and Hall, 2008; Meffert et al., 2016). This reduction in brain recruitment to perform the SWCT may be due to the intensive meditation training during the retreat. These changes may have allowed the participants from both groups to execute attentional tasks more efficiently (Moore and Malinowski, 2009; Malinowski et al., 2017).

### Between Group Analysis

In the comparison between groups, as presented in the time × group ANOVA interaction, the results highlight that the brain function of meditators and non-meditators are different before and after the retreat.

Similarly to our hypothesis based on our previous results (Kozasa et al., 2012), in this comparison between groups, after meditation retreat compared to before, in the contrast INC > NEU, non-meditators presented a reduced activation in clusters encompassing anterior cingulate/ventromedial prefrontal cortex, putamen/pallidum/caudate/temporal lobe (center), insula/putamen/temporal lobe (right) and the posterior cingulate—regions related to attention and inhibition control. Most of these regions are also activated during meditation regardless the style of the practice according to a meta-analysis about functional neuroimaging of meditation (Fox et al., 2016), therefore the SWCT can be considered an interesting task to evaluate meditation effects.

Due to the non-meditators intensive training, a neuroplastic adaptation possibly allowed their brains to become more efficient in a task which requires attention such as the SWCT (less brain activation and same behavioral response, from before to after the retreat). It is interesting to notice that, after the retreat, their brain activation became similar to that of the meditators before the retreat during the SWCT (Figures 2, 3).

Meditators presented reduced brain activity compared with non-meditators before the retreat in these regions, which might be attributed to their previous training in meditation, since it facilitates maintaining attention to the task (Kozasa et al., 2012). On the other hand, meditators showed an increase in brain activation in these regions after the same training.

### Potential Mechanisms and Brain Regions

Meditation increases our ability to intentionally bring our attention to the internal and external experiences in the present moment (Baer, 2003). A meditator is trained to experience his/her everyday life focused on the present moment with curiosity. Enhanced perception of the present moment as well as attention and awareness, including in body sensations are more often reported by more experienced meditators when compared to less experienced meditators after a meditation retreat (Kozasa et al., 2015). These changes might be related to the activation of salience network regions as well as regions connected to them presented in our group × time interaction (Figure 2 and Table 3). They are involved in the orientation of attention to the most relevant (salient) ongoing internal and external events. They may play a causal role in switching between the Central Executive Network (CEN) and the DMN, two networks with competitive interactions across tasks and stimulus, considered to mediate attention to the external and internal worlds (Bressler and Menon, 2010).

In a previous study evaluating brain activity during meditation focused on breathing, the salience network activation was associated with the moment when the meditator perceived the awareness of mind-wandering and pressed a button (Hasenkamp and Barsalou, 2012). While performing the entire SWCT in our experiment, the subject had to press a button choosing the colors of the words, therefore having to keep awareness of each moment of the task, as well as monitoring and controlling the impulse to press the wrong button. In another study about the connectivity during the cycle of FA to breathing (mind wandering, awareness of mind wandering, shifting of attention and sustained attention), one of the seeds consisted of the DLPFC. It was derived from activations during sustained attention (Hasenkamp et al., 2012). The functional connectivity of this region to the right insula, one of the regions activated more in meditators in our study, after the retreat, was stronger in high practice meditators compared to low practice, a result that might explain the increased present moment awareness of experienced meditators (Farb et al., 2007; Craig, 2009).

Some studies suggest that meditation practice is associated with changes in the anterior cingulate cortex, insula, temporo-parietal junction, fronto-limbic network and DMN structures (such as the posterior cingulate). These regions are involved in awareness, attention and emotion regulation converging to enhance self-regulation (Hölzel et al., 2011; Vago and Silbersweig, 2012; Chiesa et al., 2013; Sun et al., 2015; Tang et al., 2015). It might help to explain the differences found in the group*time interaction in our results. Meditators compared to non-meditators, may have a better self-regulation strategy, especially after the retreat that allows them to increase their focus on the task.

Brefczynski-Lewis et al. (2007) compared meditators with three levels of expertise (beginners; 19,000 h; and 44,000 h of practice) reporting an evolution in brain activation in which the most experienced meditators presented a similar brain activation pattern as compared to the beginners in attention related regions. In between groups analysis, our non-meditators after the retreat had a similar brain activation to meditators before the retreat. On the other hand, meditators had a similar brain activation after the retreat compared to non-meditators before, in the between groups analysis. This may be due to differences in strategy related to the differences in expertise, brain mechanisms involved as well as neuroplasticity.

In general, as seen in the within group analysis (Supplementary Figure S1), meditators had a decrease in brain activation during the SWCT comparing before to after the retreat. However, when compared to non-meditators there is an increase in regions which are more related to the salient or alert focus (Figures 2, 3). Therefore, the meditation retreat possibly evoked, in the meditators group, a general decrease in mental effort, however with a higher alert focus compared to non-meditators. Experienced meditators maintain attention with less effort than beginners associated with higher alert focus and clarity of the mind according to traditional texts (Wallace, 2006).

No significant results were found between meditators and non-meditators regarding the number of correct responses and RT during the incongruent condition comparing groups and before and after the retreat. These results are not according to previous findings (Chan and Woollacott, 2007; Fan et al., 2015), and may be due to the high educational level of the sample, which have led to a ceiling effect on the task (Bruyer et al., 1995).

### Limitations and Considerations for Future Studies

A limitation of the method is that it did not allow us to determine if the enhanced performance could be due to a neural mechanism or a vascular one, as noticed in an fNIRS study in which meditation increased cerebral oxygenation and enhanced performance, associated with activation of the PFC (Deepeshwar et al., 2015). Moreover, as physiological noise represents an important confound in fMRI studies (Birn et al., 2006), we believe that future works that include these measures would provide more insight into the nature of the results observed. The blocks of the Stroop task were not counterbalanced and it could have influenced the results. Another limitation of the study is the small number of participants and the reduced number of women in the study, however it reflects the profile of meditation practitioners with at least 3 years of regular practice in the region of the study. Future studies would present control groups (waiting lists of participants) for both, meditators and non-meditators. Considering case studies and case series about possible adverse effects of intensive meditation practices for patients with severe psychiatric disorders, we recommend caution in the screening of participants (Van Dam et al., 2018).

## Conclusion

As far as we know, this is the first study comparing the effects of a well-controlled meditation retreat environment on the brain of non-meditators and regular meditators.

Non-meditators, after the retreat, presented reduced brain activation during the attention and inhibition control task, becoming similar to meditators before the retreat, which points to greater efficiency learned, in only 7 days. Meditators, on the other hand, presented a general reduction in brain activation during the task, after the retreat, in within group analysis; however, compared to non-meditators, they had an increase in regions related to salience or alert focus. Therefore, intensive meditation training (retreat) presented distinct effects on the attention-related regions in meditators and non-meditators probably due to differences in expertise, attention processing as well as neuroplasticity.

## Author Contributions

EK: conception and design of the work; acquisition of data; interpretation; revision and final approval of the article; agreement to be accountable for all aspects of the work. JB, JRS, KTC and SL: analysis; revision and final approval of the article; agreement to be accountable for all aspects of the work. JR: interpretation; revision and final approval of the article; agreement to be accountable for all aspects of the work. LEM and EA: conception and design of the work; analysis and interpretation of data; revision and final approval.

## Conflict of Interest Statement

The authors declare that the research was conducted in the absence of any commercial or financial relationships that could be construed as a potential conflict of interest.

## Footnotes

^1^http://www.nordicneurolab.com

^2^http://www.fmrib.ox.ac.uk/fsl

## Supplementary Material

FIGURE S1 Within group brain activation in meditators and non-meditators. Non-meditators: clusters encompassing posterior cingulate gyrus, caudate, occipital lobe and frontal pole presented reduced activation after the retreat. Meditators: clusters encompassing middle frontal gyrus, parietal lobe, inferior frontal gyrus (IFG) and superior parietal lobule showed reduced activation after the retreat.
