# Supplementary figures and images for: Effects of a 7-Day Meditation Retreat on the Brain Function of Meditators and Non-Meditators During an Attention Task
Source: Front Hum Neurosci. 2018 Jun 11;12:222. doi: 10.3389/fnhum.2018.00222 (PMC6004402; doi:10.3389/fnhum.2018.00222)

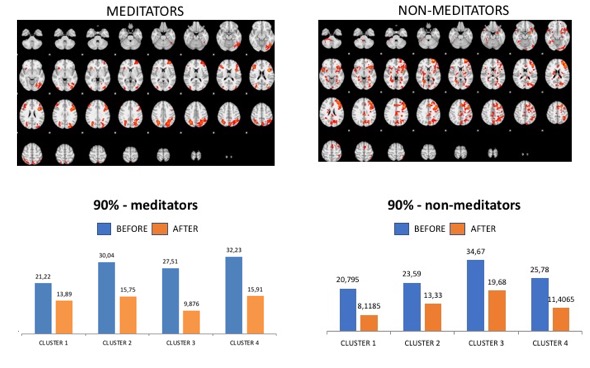

Supplement: FIGURE S1 — Within group brain activation in meditators and non-meditators. Non-meditators: clusters encompassing posterior cingulate gyrus, caudate, occipital lobe and frontal pole presented reduced activation after the retreat. Meditators: clusters encompassing middle frontal gyrus, parietal lobe, inferior frontal gyrus (IFG) and superior parietal lobule showed reduced activation after the retreat. [file Image_1.JPEG]
